# Data regarding active psychosis and functional outcome, among other clinical variables, during early phases of the illness in first-episode psychosis in the PAFIP 10-year follow-up program

**DOI:** 10.1016/j.dib.2020.105599

**Published:** 2020-04-23

**Authors:** Guillermo Pardo-de-Santayana, Javier Vázquez-Bourgon, Marcos Gómez-Revuelta, Rosa Ayesa-Arriola, Victor Ortiz-Garcia de la Foz, Benedicto Crespo-Facorro, José María Pelayo-Terán, the PAFIP research group

**Affiliations:** a Department of Psychiatry, Marqués de Valdecilla University Hospital, IDIVAL, CIBERSAM, School of Medicine, University of Cantabria, Santander, Spain; b CIBERSAM, Virgen del Rocio University Hospital-IBIS, Seville University; c Department of Psychiatry and Mental Health, El Bierzo Hospital, GASBI, SACYL, CIBERSAM, Ponferrada, León, Spain

**Keywords:** Schizophrenia, First-episode, Early intervention, Psychosis, Functional outcome, Neurotoxicity hypothesis

## Abstract

This article describes data related to the research study entitled “Duration of active psychosis during early phases of the illness and functional outcome: The PAFIP 10-year follow-up study.” [1]. We present data concerning the clinical and sociodemographic characteristics of a sample of drug-naïve patients with a first episode of non-affective psychosis. The dataset was obtained from a 3-year longitudinal intervention program as part of an ongoing 10-year epidemiological study. The tables and figure shown present the data from the analysis between the active psychosis (presence of positive psychotic symptoms), among other sociodemographic and clinical predictor variables, recorded during the 3-year longitudinal intervention program and the evaluation of the functional outcome (social functioning and functional recovery) present at the 10-year mark. The data explores how those early parameters could influence long-term outcome.

Specifications Table

**Table:** 

Subject	Psychiatry and Mental Health.
Specific subject area	Long-term outcome in first-episode psychosis.
Type of data	Tables and a figure.
How data were acquired	Prospective observational analytical study, including clinical evaluation.
Data format	Raw and analyzed.
Parameters for data collection	Drug-naïve patients with a first-episode of non-affective psychosis were included in a prospective observational analytical study (PAFIP) and treated with antipsychotics. Clinical examinations were carried out prospectively during a 3-years follow-up period. A clinical and functional assessment was carried out after a 10-year period following inclusion in program.
Description of data collection	Psychotic symptoms were measured employing the 24-item Brief Psychiatric Rating Scale (BPRS), the Scale for the Assessment of Positive Symptoms (SAPS), the Scale for the Assessment of Negative Symptoms (SANS), the premorbid adjustment scale (PAS) and the Clinical Global Impression (CGI), among other clinical and sociodemographic surveys. Patients were seen frequently in our outpatient clinic and were granted rapid access if clinical exacerbations. Moreover, thorough examinations were carried out at baseline, 3 weeks, 6 weeks, 3 months, 6 months, 12 months, 18 months, 24 months, 30 months, 36 months and 10 years.
Data source location	Autonomous region of Cantabria, Spain.
Data accessibility	Repository name: Mendeley Data Data identification number: 10.17632/2crz5nd4w3.1 Direct URL to data: http://dx.doi.org/10.17632/2crz5nd4w3.1
Related research article	Pardo-de-Santayana G, Vázquez-Bourgon J, Gómez-Revuelta M, Rosa Ayesa-Arriola R, Ortiz-Garcia de la Foz V, Crespo-Facorro B, Pelayo-Terán JM. (2020). Duration of active psychosis during early phases of the illness and functional outcome: The PAFIP 10-year follow-up study. *Schizophrenia Research.*https://doi.org/10.1016/j.schres.2020.03.009

## Value of the data

• Exploring how early clinical and sociodemographic factors influence long-term social functionality and functional recovery in first-episode psychosis could increase our understanding of how improve their long-term outcome.
• These data could benefit all those interested in treating or studying psychosis and how it affects the long-term outcome of the patients who suffer it.
• These data could be used for analyzing the progression of the clinical characteristics of patients undergoing a first-episode psychosis.

## 1 Data description

We show in this article data derived from a study on the relation between active psychosis (presence of positive psychotic symptoms), among other sociodemographic and clinical variables recorded during the 3-year longitudinal intervention program, and a cross-sectional measurement of the functional outcome (social functioning and functional recovery) present 10-years after inclusion. These data were collected form a Spanish prospective observational analytical study of drug-naïve patients with a first episode of non-affective psychosis [1]. Raw data has been made accessible through the public data repository “Mendeley Data” at http://dx.doi.org/10.17632/2crz5nd4w3.1

Table 1 describes the data of a logistic regression model for social functioning. The model (χ²: 42.091; p<0.001; Nagelkerke R2: 0.335) included DAP (Duration of Active Psychosis; that measures the time from the initiation of positive psychotic symptomatology till its remission), mean PAS (Premorbid Adjustment Scale), initial BPRS (Brief Psychiatric Rating Scale), initial negative dimension, gender, hospitalization, educational level, unemployment at onset, active at onset and diagnosis at 6 months. Mean PAS, initial BPRS and gender were the significant predictors. Mean PAS was the main predictor of the logistic regression model (Wald: 17.560; p < 0.001).

**Table 1:** Logistic Regressions for Social Functioning

Table 2 describes the data of a logistic regression model for functional recovery. The model (χ²: 14.542; p=0.002; Nagelkerke R2: 0.124) included DAP, mean PAS, hospitalization, socioeconomic level, educational level, active at onset and diagnosis at 6 months. None were significant predictors.

**Table 2:** Logistic Regressions for Functional Recovery

**Fig. 1:**
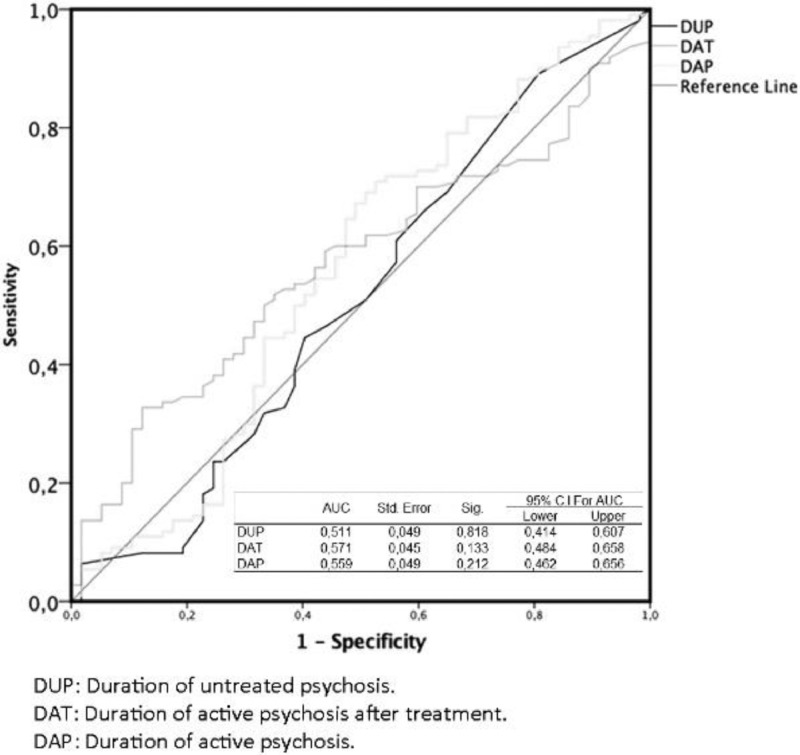
ROC Curves for Functional Recovery

Fig. 1 shows the ROC curves of DUP (Duration of Untreated Psychosis), DAT (Duration of psychosis After Treatment) and DAP (Duration of Active Psychosis) ROC curves for poor functional recovery. None of the AUC were statistically significant.

## 2 Experimental design, materials, and methods

### 2.1 Population description

To obtain these data we included in the analysis neuroleptic naïve adult patients with a first-episode of non-affective psychosis from February 2001 to July 2008. They were included in a 3-year longitudinal intervention program as part of an ongoing 10-year epidemiological study for first-episode psychosis (PAFIP) being conducted at the inpatient unit and outpatient clinic at the University Hospital Marques de Valdecilla, Spain. A detailed account of this program has been described in a previous article [2].

Only after written informed consent were patients incorporated to the PAFIP program. The program submits to the Declaration of Helsinki and was authorized by the local Institutional Review Board, in compliance with international standards for research ethics.

In the prospective observational analytical study from where these data were collected, we assessed the relationship of functional outcome with DUP (Duration of Untreated Psychosis), DAT (Duration of psychosis After Treatment) and DAP (Duration of Active Psychosis) in patients with first-episode non-affective psychosis. During a 3-year clinical follow-up, psychiatrists assessed patients in a regular basis. Psychotic symptoms were rated employing the (SAPS) [3], (SANS) [4] and (BPRS) [5]. The CGI scale was used to determine the evolution and severity of the symptoms [6]. Patients were seen frequently, based on their clinical status from weekly to quarterly visits and had easy access to appointments in the event of the appearance of any symptom. Also, evaluations were carried out at baseline, 3 weeks, 6 weeks, 3 months, 6 months, 12 months, 18 months, 24 months, 30 months and 36 months. After a 10-year period since inclusion in program nother assessment was carried out.

The duration of untreated psychosis (DUP) was defined as the time from the emergence of first continuous psychotic symptoms to introduction of adequate antipyschotic treatment. The date of the onset of the psychotic symptomatology was defined as the moment when first continuous psychotic symptom appeared and was estimated using information from patients, relatives and clinical records. To certify a systematically reliable measurement of the date of psychotic onset, we gathered information through a semi-structured interview, elicited on the Symptom Onset in Schizophrenia (SOS) inventory [7] and SCID deducing the date using the total SAPS score ≥ 3 as the threshold.

The duration of active psychosis after treatment (DAT) was defined as the amount of time, during the 3-year clinical follow-up, that a patient experienced active psychosis (positive psychotic symptomatology) after initiation of antipsychotic medication. We recorded the amount of time where patients had a score ≥ 3 on any of the four SAPS items. The duration of any psychotic relapse or clinical exacerbation was added. Psychotic relapses were recorded in patients who had previously achieved clinical remission (CGI rating ≤4, a reduction ≥ 30% on BPRS total score and having all BPRS key symptom items rated ≤3 for more than 4 consecutive weeks during) [8] and was defined by any of the following criteria for at least 1 week of duration [9], [10]: (1) a rating of ≥ 5 on any key BPRS symptom items (2) CGI rating ≥ 6 and a change in the CGI score of “much worse” or “very much worse”; (3) hospitalization for psychotic psychopathology; (4) suicide [2], [11]. Clinical exacerbations were defined as a 2-point increase on any of the key BPRS symptoms (unusual thought content, hallucinations, suspiciousness, conceptual disorganization and bizarre behavior), excluding all the cases where the rating remained at the nonpsychotic level.

Duration of Active Psychosis (DAP) was calculated by adding DUP and DAT for each patient of the study.

The measured sociodemographic variables were: premorbid adjustment scale (PAS) with the recommendations of van Mastrigt and Addington for early psychosis [12] at childhood, early and late adolescence, adulthood and the year before the onset of illness; occupational status for 2 years prior to the initial interview (1. Employment/student; 2. Unemployed); housing arrangements at the onset of psychosis (1. Living with relatives; 2. Living alone and other status); gender; educational level (1. Primary education; 2. 10-years of education or higher).

Social functioning was measured with the the Global Evaluation (GE) of the Disability Assessment Scale (DAS) [13] in the 10-year evalutation. Good social functioning was identified as a score ≤ 1 on the GE, while a score ≥ 2 on the GE was considered poor social functioning.

Functional recovery was measured in the 10-year evalutation and was defined as being in a part-time/full-time job or studying while concomitantly having reached social functioning during the at least the last 6 onths [2], [3], [4], [5], [6], [7], [8], [9], [10], [11], [12], [13], [14]

## 3 Analysis

Firstly we run a descriptive univariate analysis of social functioning and functional recovery as dichotomous variables (good/poor social functioning and good/poorfunctional recovery).

Secondly we conducted logistic regression models to predict social functioning and functional recovery using DAP, together with the other significant predictors from the univariate analysis were included at the start.

We used receiver operating characteristic (ROC) curves to determine the area under the curve (AUC) for DUP, DAT and DAP to estimate the capacity of theses variables to discriminate true positives and false positives for poor functional recovery.

The analysis was completed with SPSS, version 19.0 (SPSS Inc., Chicago, IL, USA). The statistical significance was set at 0.05 and in all cases we used two-tailed statistical test.
